# Evidential MACE prediction of acute coronary syndrome using electronic health records

**DOI:** 10.1186/s12911-019-0754-7

**Published:** 2019-04-09

**Authors:** Danqing Hu, Wei Dong, Xudong Lu, Huilong Duan, Kunlun He, Zhengxing Huang

**Affiliations:** 1 College of Biomedical Engineering and Instrument Science, Zhejiang University, Key Lab for Biomedical Engineering of Ministry of Education, Zheda Road, Hangzhou, China; 2 Department of Cardiology, Chinese PLA General Hospital, Fuxing Road, Beijing, China; 3 Beijing Key Laboratory Of Chronic Heart Failure Precision Medicine, Beijing, China

## Abstract

**Background:**

Major adverse cardiac event (MACE) prediction plays a key role in providing efficient and effective treatment strategies for patients with acute coronary syndrome (ACS) during their hospitalizations. Existing prediction models have limitations to cope with imprecise and ambiguous clinical information such that clinicians cannot reach to reliable MACE prediction results for individuals.

**Methods:**

To remedy it, this study proposes a hybrid method using Rough Set Theory (RST) and Dempster-Shafer Theory (DST) of evidence. In details, four state-of-the-art models, including one traditional ACS risk scoring model, i.e., GRACE, and three machine learning based models, i.e., Support Vector Machine, *L*_1_-Logistic Regression, and Classification and Regression Tree, are employed to generate initial MACE prediction results, and then RST is applied to determine the weights of the four single models. After that, the acquired prediction results are assumed as basic beliefs for the problem propositions and in this way, an evidential prediction result is generated based on DST in an integrative manner.

**Results:**

Having applied the proposed method on a clinical dataset consisting of 2930 ACS patient samples, our model achieves 0.715 AUC value with competitive standard deviation, which is the best prediction results comparing with the four single base models and two baseline ensemble models.

**Conclusions:**

Facing with the limitations in traditional ACS risk scoring models, machine learning models and the uncertainties of EHR data, we present an ensemble approach via RST and DST to alleviate this problem. The experimental results reveal that our proposed method achieves better performance for the problem of MACE prediction when compared with the single models.

## Background

Acute coronary syndrome (ACS) refers to a group of conditions due to decreased blood flow in the coronary arteries such that part of the heart muscle is unable to function properly or dies [1, 2]. Major adverse cardiac events (MACE) indicates the composite of a variety of adverse events related to the cardiovascular system [3, 4], which may lead severe or fatal outcome for ACS patients. MACE prediction, as a crucial and widely explored topic, plays a pivotal role in the optimal management for ACS patients at their early stage of hospitalization, e.g., clinical decision making of care and treatment, drug development and cost estimation [4, 5].

Over the past decades, a mountain of studies has been proposed to facilitate risk assessment [1, 4]. Many traditional ACS risk score tools, e.g., TIMI [5], PURSUIT [6] and GRACE [7], have been widely used in real clinical circumstances and shown good discriminatory accuracy in predicting MACE for ACS patients [8, 9]. However, these traditional models have several inherent limitations [10]. In particular, these models developed using data from clinical trials and registries may be not representative of a general department patient population because there are strict inclusion and exclusion criteria of the cohort [1]. In addition, to obtain a simple and easy-use tool, traditional risk scoring models are established on a small set of handy-picked risk factors based on the significant univariate relationship to the end point by univariate logistic regression, which may cause deterioration of predicting performance [4, 10, 11]. Moreover, it is hard to enroll new and more discriminatory risk factors into those traditional models, which limits their extension ability [1].

Recently, with the rapid growth of electronic health records (EHRs) data, a multitude risk prediction models utilizing the potential of EHRs have become available and achieved significant improvements in this field [4, 10–13]. Most of these models are built based on machine learning and data mining techniques. Although valuable, there are still some deficiencies to apply them on mining EHRs, particularly due to the vagueness, impreciseness and uncertain clinical information contained in EHR data. Specifically, most of these models assume that MACEs have been correctly annotated in the EHR dataset and the focus is on the learning capabilities of the MACE prediction scheme. However, unambiguous MACE annotations may be difficult and imprecise due to the lack of information required for specifying certain MACE labels to patient individuals.

Both the traditional risk scoring models and machine learning based models provide us with diverse perspectives on the problem of MACE prediction [4], so that each of them results in complementary information and could be fused to produce an integrative and reliable result. By utilizing a proper strategy for the construction of an ensemble network, it can be successfully applied to MACE prediction problem with imprecise and uncertain information. Dempster-Shafer Theory [14, 15] (DST) of evidence is a general framework for reasoning with uncertainty by combining multiple evidences together to obtain a more reliable result, which has been widely employed in sensor fusion [16], financial distress detection [17], medical diagnosis [18] and etc. To this end, we propose a hybrid method using Rough Set Theory [19] (RST) and Dempster-Shafer Theory of evidence for MACE prediction. The proposed approach integrates four state-of-the-art models, including one traditional ACS risk scoring model, i.e., GRACE, and three machine learning based models, i.e., Support Vector Machine [20] (SVM), *L*_1_-Logistic Regression [21] (*L*_1_-LR), and Classification and Regression Tree [22] (CART), to generate comprehensive and reliable MACE prediction results. In particular, RST is applied to determine the weights of the four single models, and then the prediction results generated by these single models are assumed as basic beliefs for the problem propositions and in this way, an ensemble MACE prediction result is generated by combine each single model’s evidence such that the overall prediction performance can be enhanced.

We comparatively evaluate the performance of the proposed model on a clinical dataset consisting of 2930 ACS patients and collected from the cardiology department of Chinese PLA General Hospital. The experimental results demonstrate that, in terms of reducing uncertainty caused human subjective cognition on patient data recording and annotation, our proposed method performs better than traditional single models.

## Preliminaries

### Rough set theory

Rough set theory was first proposed by Pawlak [19], which is widely used to deal with problem containing uncertainty. In RST, an information system is defined as a pair $\mathbb{I}=\left(\mathrm{U},\mathrm{A}\cup \mathrm{R}\right)$, where U = {u_1_, u_2_,  … , u_t_} is a nonempty set of finite objects, A = {a_1_, a_2_,  … , a_n_} is a nonempty set of finite attributes, R = {r_1_, r_2_,  … , r_m_} is a nonempty set of finite results. With each subset P ⊆ A, there is an indiscernibility relation (also called equivalence relation) defined asIND(P) = {(x, y) ∈ U^2^| ∀a_i_ ∈ P, a_i_(x) = a_i_(y)}. The set of objects U can be partitioned based on the relation IND(P), which is denoted by U ∕ IND(P), where an element from U ∕ IND(P) is called an equivalence class. According to equation above, the indiscernibility relation of A, R, and A − {a_j_}, are defined as IND(A) = {(x, y) ∈ U^2^| ∀a_i_ ∈ A, a_i_(x) = a_i_(y)}, IND(R) = {(x, y) ∈ U^2^| ∀r_i_ ∈ R, r_i_(x) = r_i_(y)}, and IND(A − {a_j_}) = {(x, y) ∈ U^2^| ∀a_i_ ∈ A, a_i_ ≠ a_j_, a_i_(x) = a_i_(y)}, j = 1, 2, … , m. Depending on the theory of entropy, the dependence of R to A can be defined as:

1 $$ \mathrm{D}\left(\mathrm{IND}\left(\mathrm{R}\right)/\mathrm{IND}\left(\mathrm{A}\right)\right)=-\sum \limits_{\left[\mathrm{x}\right]\in \mathrm{U}/\mathrm{IND}\left(\mathrm{R}\right)}\mathrm{p}\left[\mathrm{x}\right]\sum \limits_{\left[\mathrm{y}\right]\in \mathrm{U}/\mathrm{IND}\left(\mathrm{A}\right)}\mathrm{p}\left(\left[\mathrm{y}\right]/\left[\mathrm{x}\right]\right)\ln \left(\mathrm{p}\left(\left[\mathrm{y}\right]/\left[\mathrm{x}\right]\right)\right) $$

where $\mathrm{p}\left[\mathrm{x}\right]=\frac{\operatorname{card}\left[\mathrm{x}\right]}{\operatorname{card}\left[\mathrm{U}\right]}$, $\mathrm{p}\left(\left[\mathrm{y}\right]/ \left[\mathrm{x}\right]\right)=\frac{\operatorname{card}\left(\left[\mathrm{y}\right]\cap \left[\mathrm{x}\right]\right)}{\operatorname{card}\left[\mathrm{x}\right]}$. The significance of attribute a_j_ can be defined as:

2 $$ \upomega \left({\mathrm{a}}_{\mathrm{j}},\mathrm{A},\mathrm{R}\right)=\left|\mathrm{D}\left(\mathrm{IND}\left(\mathrm{R}\right)/\mathrm{IND}\left(\mathrm{A}-\left\{{\mathrm{a}}_{\mathrm{j}}\right\}\right)\right)-\mathrm{D}\left(\mathrm{IND}\left(\mathrm{R}\right)/\mathrm{IND}\left(\mathrm{A}\right)\right)\right|,\mathrm{j}=1,2,\dots, \mathrm{m}. $$

Finally, the weight of attribute a_j_ is defined as follows:

3 $$ \mathrm{w}\left({\mathrm{a}}_{\mathrm{j}}\right)=\frac{\upomega \left({\mathrm{a}}_{\mathrm{j}},\mathrm{A},\mathrm{R}\right)}{\sum \limits_{\mathrm{j}=1}^{\mathrm{m}}\upomega \left({\mathrm{a}}_{\mathrm{j}},\mathrm{A},\mathrm{R}\right)} $$

### Dempster-Shafer theory

Let Θ be the frame of discernment, which represents all possible mutually exclusive states of a system. The power set 2^Θ^ is the set of all subset of Θ, including the empty set ∅, which represents propositions related to actual state of the system. The basic probability assignment (BPA) is defined as m : 2^Θ^ → [0, 1], where m satisfies: m(∅) = 0, $\sum \limits_{\mathrm{A}\subseteq \mathrm{X}}\mathrm{m}\left(\mathrm{A}\right)=1$ and m(A) is called BPA of proposition A. If m(A) > 0, the subset A is called focal element. The belief function of proposition A denoted as Bel(A) is defined as $\mathrm{Bel}\left(\mathrm{A}\right)=\sum \limits_{\mathrm{B}\subseteq \mathrm{A}}\mathrm{m}\left(\mathrm{B}\right),\forall \mathrm{A}\subseteq \Theta$. The plausibility function of proposition A denoted as Pl(A) is defined as $\mathrm{Pl}\left(\mathrm{A}\right)=1-\mathrm{Bel}\left(\overline{\mathrm{A}}\right)=\sum \limits_{\mathrm{B}\cap \mathrm{A}\ne \varnothing}\mathrm{m}\left(\mathrm{B}\right),\forall \mathrm{A}\subseteq \Theta .$ The belief function and plausibility function represent the minimal and maximal support of A based on the BPA, respectively.

When the system has more than one basic probability assignment functions, Dempster’s combinational rule can combine them together. Let m_1_ and m_2_ be the two different BPA functions, and the evidences are A_1_, A_2_, … , A_m_ with respect to m_1_ and B_1_, B_2_, … , B_n_ with respect to m_2_, if $\sum \limits_{{\mathrm{A}}_{\mathrm{i}}\cap {\mathrm{B}}_{\mathrm{j}}=\varnothing }{\mathrm{m}}_1\left({\mathrm{A}}_{\mathrm{i}}\right){\mathrm{m}}_2\left({\mathrm{B}}_{\mathrm{j}}\right)<1$, we have:

4 $$ {\mathrm{m}}_{1,2}\left(\mathrm{C}\right)={\mathrm{m}}_1\bigoplus {\mathrm{m}}_2\left(\mathrm{A}\right)=\left\{\begin{array}{c}\frac{1}{1-\mathrm{K}}\sum \limits_{{\mathrm{A}}_{\mathrm{i}}\cap {\mathrm{B}}_{\mathrm{j}}=\mathrm{C}}{\mathrm{m}}_1\left({\mathrm{A}}_{\mathrm{i}}\right){\mathrm{m}}_2\left({\mathrm{B}}_{\mathrm{j}}\right),\forall \mathrm{C}\subseteq \Theta, \mathrm{C}\ne \varnothing \\ {}0,\mathrm{C}=\varnothing \end{array}\right. $$

where $\mathrm{K}=\sum \limits_{{\mathrm{A}}_{\mathrm{i}}\cap {\mathrm{B}}_{\mathrm{j}}=\varnothing }{\mathrm{m}}_1\left({\mathrm{A}}_{\mathrm{i}}\right){\mathrm{m}}_2\left({\mathrm{B}}_{\mathrm{j}}\right)$, which indicates the conflict between the evidences, called conflict probability. And the coefficient $\frac{1}{1-\mathrm{K}}$ is a normalization factor.

## Methods

**Fig. 1:**
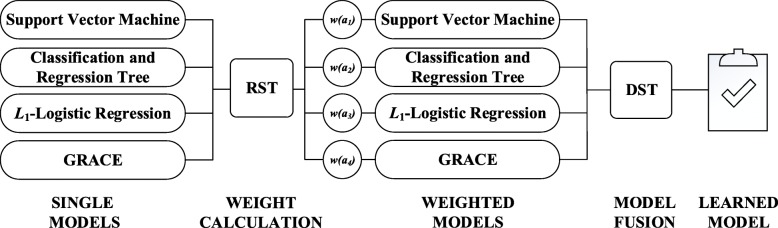
The outline of the proposed method

In this study, we propose an ensemble approach to integrate traditional risk scoring models and advanced machine learning based models together to alleviate the limitations we mentioned above. Figure 1 shows the outline of our proposed method. As depicted in Fig. 1, we firstly calculated the weights for the four single models, i.e., GRACE, SVM, CART, and *L*_1_-LR, based on RST. After that, we employed the DST to integrate the weighted outputs of each model together as our ensemble MACE prediction result.

To give a more understandable explanation for our proposed method, we employed a subset of our real world dataset to show how we implemented our method step by step. Table 1 shows 10 patient samples from the collected dataset with their corresponding outputs from models trained in our previous work.

**Table 1:** The original outputs of single models for 10 patient samples

Instances	Single Models				Actual MACE results
	SVM	*L*_1_-LR	CART	GRACE	
1	0.7434	0.6032	0.6716	201	Y
2	0.1250	0.1884	0.1890	85	N
3	0.2651	0.1798	0.1890	56	N
4	0.1735	0.3272	0.3277	92	N
5	0.1608	0.3347	0.3277	119	N
6	0.7260	0.6531	0.6716	132	N
7	0.1601	0.3104	0.3346	133	Y
8	0.1171	0.1927	0.1890	137	N
9	0.4829	0.3041	0.3346	92	Y
10	0.1743	0.2050	0.3277	97	N

### Weights calculation using rough set theory

Before calculating the weight of each single prediction model, we need to transform the models’ outputs into dichotomous variables, such that we can apply RST to calculate the dependence of each model to the final prediction results. We choose the output that is closest to the top-left point in the area under the curve (AUC) figure as our threshold to transform the model’s outputs. Experimentally on all patient samples we have, the thresholds are 0.2348, 0.22689, 0.2584 and 106.5 for SVM, *L*_1_-LR, CART and GRACE, respectively. We tend to use the data obtained from our work to give a more practical description in this and following sections. According to the dichotomized outputs, we can calculate the weight for each single model based on Eq. (1–3). The weights are 0.5363, 0.1765, 0.1177 and 0.1696 for SVM, *L*_1_-LR, CART and GRACE. Table 2 shows the dichotomized outputs, optimal thresholds and weights of the 4 single models.

**Table 2:** The dichotomized outputs, optimal thresholds and weights of single models for 10 patient samples

Instances	Single Models				Actual MACE results
	SVM	*L*_1_-LR	CART	GRACE	
1	1	1	1	1	1
2	0	0	0	0	0
3	1	0	0	0	0
4	0	1	1	0	0
5	0	1	1	1	0
6	1	1	1	1	0
7	0	1	1	1	1
8	0	0	0	1	0
9	1	1	1	0	1
10	0	0	1	0	0
Threshold	0.2348	0.2689	0.2584	106.5	NA
Weight	0.5363	0.1765	0.1177	0.1696	NA

### Model fusion using Dempster-Shafer evidence theory

Before using the Dempster-Shafer Theory to combine the four models’ outputs together, we need to transform the models’ outputs into basic probability assignments (BPA). However, in our study, we notice that the range of GRACE’s outputs is from 2 to 258, which cannot be directly used as the BPA, and moreover, the four single models we employed have different optimal thresholds which may influence the combination results. To alleviate these problems, we first normalize the GRACE’s outputs to between 0 and 1 by Eq. (5), and then apply Eq. (6) to adjust the threshold of each single model to the same value, i.e. 0.5, to eliminate the influence caused by different optimal thresholds.

5 $$ {\mathrm{A}}_{\mathrm{GRACE},\mathrm{j}}=\frac{{\mathrm{O}}_{\mathrm{GRACE},\mathrm{j}}-{\min}_{\mathrm{GRACE}}}{\max_{\mathrm{GRACE}}-{\min}_{\mathrm{GRACE}}};\mathrm{j}=1,2,3,\dots, \mathrm{n} $$

where n is the number of patients, O_GRACE, j_ and A_GRACE, j_ indicate the original and normalized output of the GRACE model for the jth patient, respectively. max_GRACE_ and min_GRACE_, the maximum value and minimum value of the original output of GRACE, are 37 and 201 in our study, respectively.

6 $$ {{\mathrm{A}}^{\ast}}_{\mathrm{i},\mathrm{j}}=\left\{\begin{array}{c}\ 0.5\times \frac{{\mathrm{A}}_{\mathrm{i},\mathrm{j}}}{{\mathrm{Threshold}}_{\mathrm{i}}},{\mathrm{A}}_{\mathrm{i},\mathrm{j}}<{\mathrm{Threshold}}_{\mathrm{i}}\\ {}\ 0.5,{\mathrm{A}}_{\mathrm{i},\mathrm{j}}={\mathrm{Threshold}}_{\mathrm{i}}\\ {}0.5\times \frac{{\mathrm{A}}_{\mathrm{i},\mathrm{j}}-{\mathrm{Threshold}}_{\mathrm{i}}}{1-{\mathrm{Threshold}}_{\mathrm{i}}}+0.5,{\mathrm{A}}_{\mathrm{i},\mathrm{j}}>{\mathrm{Threshold}}_{\mathrm{i}}\end{array};\mathrm{j}=\right.1,2,\dots, \mathrm{n} $$

where A^∗^_i, j_ is the adjusted output of ith model for the jth patient with i∈{SVM, *L*_1_-LR, CART, GRACE}, Threshold_i_ is the ith model’s optimal threshold utilized in the dichotomization procedure for weights calculation using RST. Table 3 shows the adjusted outputs of each single model based on Eqs. (5, 6).

**Table 3:** The adjusted outputs of single models for 10 patient samples

Instances	Models				Actual MACE results
	SVM	*L*_1_-LR	CART	GRACE	
1	0.8323	0.7286	0.7786	1.0000	1
2	0.2663	0.3503	0.3658	0.3453	0
3	0.5198	0.3344	0.3658	0.1367	0
4	0.3695	0.5399	0.5468	0.3957	0
5	0.3424	0.5450	0.5468	0.5661	0
6	0.8210	0.7628	0.7786	0.6349	0
7	0.3409	0.5284	0.5514	0.6402	1
8	0.2494	0.3583	0.3658	0.6614	0
9	0.6621	0.5241	0.5514	0.3957	1
10	0.3712	0.3812	0.5468	0.4317	0

Based on the adjusted outputs, we can obtain the BPA for each patient. In our method, we combined the weights calculated by RST into the BPA using the following functions:

7 $$ {\mathrm{m}}_{{{\mathrm{A}}^{\ast}}_{\mathrm{i},\mathrm{j}}}\left(\varnothing \right)=0 $$

8 $$ {\mathrm{m}}_{{{\mathrm{A}}^{\ast}}_{\mathrm{i},\mathrm{j}}}(1)=\frac{{\mathrm{w}}_{\mathrm{i}}\times {{\mathrm{A}}^{\ast}}_{\mathrm{i},\mathrm{j}}}{{\mathrm{w}}_{\mathrm{i}}\times {{\mathrm{A}}^{\ast}}_{\mathrm{i},\mathrm{j}}+{\mathrm{w}}_{\mathrm{i}}\times \left(1-{{\mathrm{A}}^{\ast}}_{\mathrm{i},\mathrm{j}}\right)+1} $$

9 $$ {\mathrm{m}}_{{{\mathrm{A}}^{\ast}}_{\mathrm{i},\mathrm{j}}}(0)=\frac{{\mathrm{w}}_{\mathrm{i}}\times \left(1-{{\mathrm{A}}^{\ast}}_{\mathrm{i},\mathrm{j}}\right)}{{\mathrm{w}}_{\mathrm{i}}\times {\mathrm{F}}_{\mathrm{i},\mathrm{j}}+{\mathrm{w}}_{\mathrm{i}}\times \left(1-{{\mathrm{A}}^{\ast}}_{\mathrm{i},\mathrm{j}}\right)+1} $$

10 $$ {\mathrm{m}}_{{{\mathrm{A}}^{\ast}}_{\mathrm{i},\mathrm{j}}}\left(\Theta \right)=\frac{1}{{\mathrm{w}}_{\mathrm{i}}\times {{\mathrm{A}}^{\ast}}_{\mathrm{i},\mathrm{j}}+{\mathrm{w}}_{\mathrm{i}}\times \left(1-{{\mathrm{A}}^{\ast}}_{\mathrm{i},\mathrm{j}}\right)+1} $$

where w_i_ is the weight of the ith model with i∈{SVM, *L*_1_-LR, CART, GRACE}.

According to the weighted BPA obtained by Eqs. (7–10), we can employ the Dempster’s combinational rule to combine the four models’ BPA functions together. Based on Eq. (4), we have:

11 $$ {\mathrm{m}}_{{{\mathrm{A}}^{\ast}}_{\mathrm{all},\mathrm{j}}}(1)={\mathrm{m}}_{{{\mathrm{A}}^{\ast}}_{\mathrm{SVM},\mathrm{j}}}\bigoplus {\mathrm{m}}_{{{\mathrm{A}}^{\ast}}_{L_1-\mathrm{LR},\mathrm{j}}}\bigoplus {\mathrm{m}}_{{{\mathrm{A}}^{\ast}}_{\mathrm{CART},\mathrm{j}}}\bigoplus {\mathrm{m}}_{{{\mathrm{A}}^{\ast}}_{\mathrm{GRACE},\mathrm{j}}}(1) $$

12 $$ {\mathrm{m}}_{{{\mathrm{A}}^{\ast}}_{\mathrm{all},\mathrm{j}}}(0)={\mathrm{m}}_{{{\mathrm{A}}^{\ast}}_{\mathrm{SVM},\mathrm{j}}}\bigoplus {\mathrm{m}}_{{{\mathrm{A}}^{\ast}}_{L_1-\mathrm{LR},\mathrm{j}}}\bigoplus {\mathrm{m}}_{{{\mathrm{A}}^{\ast}}_{\mathrm{CART},\mathrm{j}}}\bigoplus {\mathrm{m}}_{{{\mathrm{A}}^{\ast}}_{\mathrm{GRACE},\mathrm{j}}}(0) $$

Thus, the final decision value for the jth patient, i.e., R_all, j_, can be simply represented as:

13 $$ {\mathrm{R}}_{\mathrm{all},\mathrm{j}}=\frac{{\mathrm{m}}_{{{\mathrm{A}}^{\ast}}_{\mathrm{all},\mathrm{j}}}(1)}{{\mathrm{m}}_{{{\mathrm{A}}^{\ast}}_{\mathrm{all},\mathrm{j}}}(0)+{\mathrm{m}}_{{{\mathrm{A}}^{\ast}}_{\mathrm{all},\mathrm{j}}}(1)} $$

Table 4 shows the patient sample’s BPA, the combined BPA and the final decision value. Note that the prediction results are determined by the optimal threshold of decision value, i.e., 0.4759, determined based on the same criteria as the dichotomization procedure. After all the procedures above, we can obtain the ensemble prediction model, which can consider the weight of each single model calculated by RST when combining the BPA by DST.

**Table 4:** The BPA, combined BPA and the final decision value for 10 patient samples

Instances	BPA	Single Models				Combined BPA	Decision value	Prediction results	Actual MACE results
		SVM	*L*_1_-LR	CART	GRACE				
1	1	0.2905	0.1093	0.0820	0.1450	0.4806	0.8631	1	1
	0	0.0585	0.0407	0.0233	0.0000	0.0762			
	Θ	0.6509	0.8500	0.8947	0.8550	0.4432			
2	1	0.0929	0.0525	0.0385	0.0501	0.1549	0.2845	0	0
	0	0.2561	0.0975	0.0668	0.0949	0.3894			
	Θ	0.6509	0.8500	0.8947	0.8550	0.4557			
3	1	0.1814	0.0502	0.0385	0.0198	0.2046	0.3785	0	0
	0	0.1676	0.0998	0.0668	0.1252	0.3360			
	Θ	0.6509	0.8500	0.8947	0.8550	0.4594			
4	1	0.1290	0.0810	0.0576	0.0574	0.2260	0.4190	0	0
	0	0.2201	0.0690	0.0477	0.0876	0.3133			
	Θ	0.6509	0.8500	0.8947	0.8550	0.4607			
5	1	0.1195	0.0817	0.0576	0.0821	0.2371	0.4404	0	0
	0	0.2296	0.0683	0.0477	0.0629	0.3012			
	Θ	0.6509	0.8500	0.8947	0.8550	0.4617			
6	1	0.2866	0.1144	0.0820	0.0921	0.4396	0.7999	1	0
	0	0.0625	0.0356	0.0233	0.0529	0.1100			
	Θ	0.6509	0.8500	0.8947	0.8550	0.4504			
7	1	0.1190	0.0793	0.0581	0.0928	0.2433	0.4523	0	1
	0	0.2301	0.0707	0.0472	0.0522	0.2947			
	Θ	0.6509	0.8500	0.8947	0.8550	0.4620			
8	1	0.0870	0.0537	0.0385	0.0959	0.1834	0.3397	0	0
	0	0.2620	0.0962	0.0668	0.0491	0.3564			
	Θ	0.6509	0.8500	0.8947	0.8550	0.4603			
9	1	0.2311	0.0786	0.0581	0.0574	0.3146	0.5839	1	1
	0	0.1180	0.0714	0.0472	0.0876	0.2242			
	Θ	0.6509	0.8500	0.8947	0.8550	0.4611			
10	1	0.1296	0.0572	0.0576	0.0626	0.2124	0.3931	0	0
	0	0.2195	0.0928	0.0477	0.0824	0.3278			
	Θ	0.6509	0.8500	0.8947	0.8550	0.4598			

## Experiments and results

Based on our previous work, we have obtained the original outputs of the four single models, e.g., SVM, *L*_1_-LR, CART and GRACE, for a total of 2930 ACS patient samples collected from the Cardiology Department of the Chinese PLA General Hospital. We employed 5-fold cross validation to construct both the four single models and our proposed model. To compare with other ensemble methods, we trained the Bagging [23] and AdaBoost [24] models by 5-fold cross validation as well. The metrics of area under the curve [25] (AUC), prediction accuracy (ACC) and their corresponding standard deviations (STD) are employed to evaluate all these models. All model constructions and statistical analyses were completed by R version 3.3.1 (The R Foundation for Statistical Computing, Vienna, Austria). Table 5 illustrates four single models’ weights in 5-fold cross validation. Tables 6 and 7 shows the AUC value and accuracy for all models in our study.

**Table 5:** The weights of single models in each fold

Folds	Models			
	SVM	*L*_1_-LR	CART	GRACE
1	0.5363	0.1765	0.1177	0.1696
2	0.2824	0.3410	0.2943	0.0823
3	0.5583	0.2189	0.1041	0.1187
4	0.4427	0.2433	0.2177	0.0962
5	0.3183	0.2988	0.2194	0.1634

**Table 6:** The AUC values of all models

Folds	Single Models				Ensemble Models		Proposed
	SVM	*L*_1_-LR	CART	GRACE	Bagging	AdaBoost	
1	0.742	0.724	0.644	0.641	0.714	0.678	0.736
2	0.696	0.715	0.664	0.629	0.688	0.701	0.713
3	0.704	0.689	0.594	0.635	0.707	0.696	0.707
4	0.682	0.702	0.604	0.640	0.706	0.686	0.700
5	0.711	0.704	0.645	0.636	0.683	0.672	0.717
Average	0.707	0.707	0.630	0.636	0.700	0.687	0.715
STD	±0.022	±0.013	±0.030	±0.005	±0.013	±0.012	±0.013

**Table 7:** The accuracy values of all models

Folds	Single Models				Ensemble Models		Proposed
	SVM	*L*_1_-LR	CART	GRACE	Bagging	AdaBoost	
1	0.715	0.725	0.693	0.625	0.674	0.630	0.724
2	0.662	0.703	0.679	0.592	0.667	0.655	0.717
3	0.635	0.684	0.715	0.560	0.659	0.671	0.674
4	0.695	0.659	0.734	0.679	0.677	0.654	0.671
5	0.676	0.677	0.696	0.601	0.676	0.689	0.686
Average	0.676	0.690	0.703	0.611	0.671	0.660	0.694
SD	±0.031	±0.025	±0.021	±0.045	±0.008	±0.022	±0.024

**Fig. 2:**
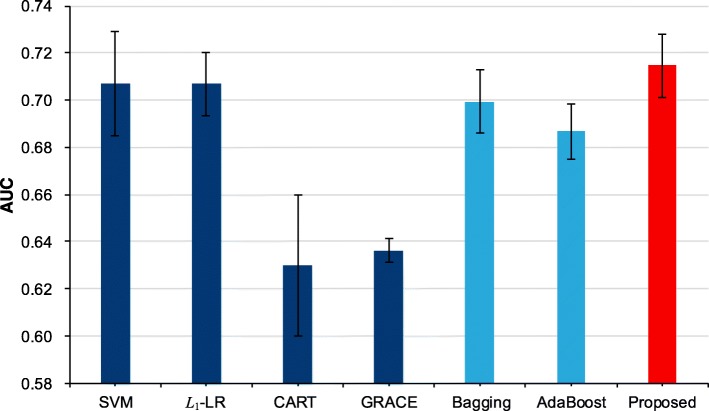
The average AUC values with standard deviation

**Fig. 3:**
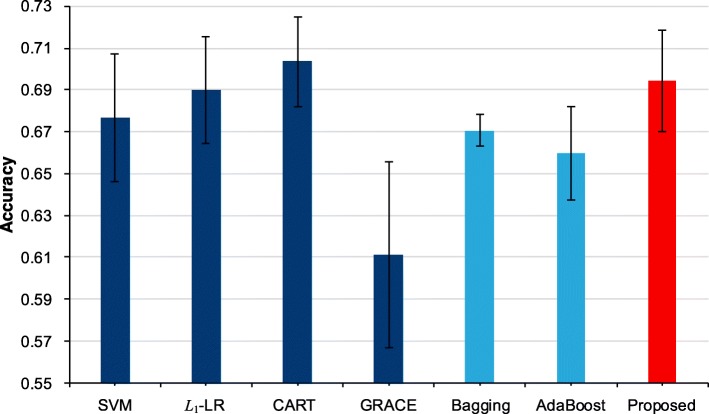
The average accuracy values with standard deviation

From Table 5, we can find that each model has different weights in each fold, which indicates that the weight calculation step in our method distinguishes the discrimination ability of each single model and affects the construction of the proposed model in each fold cross validation. As illustrated in Tables 6 and 7, we can notice that our proposed method achieves the highest AUC value comparing with the 4 single models which means it can combine the output of each single model and generate a more reliable prediction result. And also, the accuracy of our model is competitive in all models with AUC values above 0.70. Moreover, when compared with the traditional ensemble methods, i.e., Bagging and AdaBoost, our models achieve a better performance with a significant margin. Furthermore, we can notice that the proposed model is the only one whose all AUC values in 5-fold are above 0.70 with a competitive standard deviation, which indicates the outstanding stability of our method. Figures 2 and 3 presents a more understandable comparison between our proposed model and other models.

## Discussion

The problem of MACE prediction plays a vital role in the optimal treatment management for ACS patients during their hospitalizations. Facing with the limitations in traditional risk scoring models, machine learning methods and the uncertainties of EHR data, we present an ensemble approach to alleviate this problem. We firstly employed RST to determine each single MACE prediction model’s weight. And then, DST was applied to combine all weighted single models as our ensemble model so as to enhance the performance of MACE prediction. Experiments have been conducted on a clinical dataset collected from the Cardiology Department of the China PLA General Hospital. The experimental results show our proposed method achieves the best prediction performance with 0.715 AUC value, which indicates our model can combine various information provided by the single models to generate more reliable and stable prediction result on the MACE prediction problem.

It should be mentioned that there exist some problems needed further exploration.

In our current work, the single models we employed are based on our previous work directly with no further selection. However, the single model’s outputs will have a significant impact on the final prediction results. Thus, we need to explore which single models are the most appropriate for the proposed method to combine so as to improve the prediction performances. Furthermore, resampling, a key technique to construct more single models, is also a potential direction to build more powerful and robust ensemble prediction model based on the proposed method.

In our future research, we plan to develop and deploy a continuous MACE prediction service in practice. Note that the dynamic nature of a patient status is often essential to risk stratification and subsequent treatment interventions adopted in clinical practice. Thus, it would be valuable to provide a continuous MACE prediction service during patients’ length of stay. Such a service not only anticipate MACEs at runtime, but also monitors patient treatment processes in a continuous and predictive fashion.

## Conclusion

In this paper, we present an ensemble approach to alleviate the limitations in traditional ACS risk scoring models, machine learning models and the uncertainties of EHR data. We first employed RST to determine the weight for each single model. After that, DST was applied to combine the weighted outputs of single models as the final prediction results. The experimental results indicate our proposed method achieves 0.715 AUC value with a competitive standard deviation, which is a better performance for the problem of MACE prediction when compared with the single models.

## Abbreviations

ACC: Accuracy
ACS: Acute Coronary Syndrome
AUC: Area Under the Curve
BPA: Basic Probability Assignment
CART: Classification and Regression Tree
DST: Dempster-Shafer Theory
EHR: Electronic Health Record
GRACE: Global Registry of Acute Coronary Events
*L*_1_-LR: *L*_1_-Logistic Regression
MACE: Major Adverse Cardiac Event
PURSUIT: Platelet glycoprotein IIb/IIIa in Unstable angina: Receptor Suppression Using Integrilin (eptifibatide) Therapy
RST: Rough Set Theory
STD: Standard Deviation
SVM: Support Vector Machine
TIMI: Thrombolysis in Myocardial Infarction
